# Brain Implantable End-Fire Antenna with Enhanced Gain and Bandwidth

**DOI:** 10.3390/s22124328

**Published:** 2022-06-07

**Authors:** Lisa Sapari, Samnang Hout, Jae-Young Chung

**Affiliations:** 1 Department of Electrical & Information Engineering, SeoulTech, Seoul 01811, Korea; lisasapari@yahoo.com; 2 Department of Integrated IT Engineering, SeoulTech, Seoul 01811, Korea; hout.samnang22@gmail.com

**Keywords:** brain–machine interface, implantable antenna, link budget analysis, specific absorption rate, tissue-emulating phantom, ultra-wideband antenna, Vivaldi antenna

## Abstract

An end-fire radiating implantable antenna with a small footprint and broadband operation at the frequency range of 3–5 GHz is proposed for high-data-rate wireless communication in a brain–machine interface. The proposed Vivaldi antenna was implanted vertically along the height of the skull to avoid deformation in the radiation pattern and to compensate for a gain–loss caused by surrounding lossy brain tissues. It was shown that the vertically implanted end-fire antenna had a 3 dB higher antenna gain than a horizontally implanted broadside radiating antenna discussed in recent literature. Additionally, comb-shaped slot arrays imprinted on the Vivaldi antenna lowered the resonant frequency by approximately 2 GHz and improved the antenna gain by more than 2 dB compared to an ordinary Vivaldi antenna. An antenna prototype was fabricated and then tested for verification inside a seven-layered semi-solid brain phantom where each layer had similar electromagnetic material properties as actual brain tissues. The measured data showed that the antenna radiated toward the end-fire direction with an average gain of −15.7 dBi under the frequency of interest, 3–5 GHz. A link budget analysis shows that reliable wireless communication can be achieved over a distance of 10.8 cm despite the electromagnetically harsh environment.

## 1. Introduction

Brain signal monitoring has gained considerable attention not only from brain scientists but also from electronics engineers. Real-time neural data extracted by a brain–machine interface (BMI) or brain–computer interface (BCI) can be used for various applications, such as restoring sensory functions and controlling robotic prostheses [1]. A review of the basic concept of BCI, its applications, and challenges were extensively discussed in [2]. A conventional technique such as electroencephalography (EEG) requires a wired electrode to be attached on the scalp to monitor brain signals [3]. However, several studies have demonstrated the need for implanting a wireless BMI deep into the brain to monitor both the electroencephalogram (EEFG) and electrocorticography (ECoG) for cognitive and speech control [4,5,6,7,8,9]. Neuralink [10], a neurotechnology company, has recently presented a pioneering deep BMI with wireless communication and power-charging functions.

A wireless BMI consists of electrodes, an analog integrated circuit, a digital signal processing unit, a radio frequency (RF) front-end, and an antenna. They are packed into a biocompatible housing whose footprint typically ranges from 10 × 10 mm^2^ to 20 × 20 mm^2^ [5,6,7,8,9,10,11,12,13].

The slightly large size of a brain implant is primarily due to the antenna size. For instance, the size of a half-wavelength patch antenna at the 2.4 GHz industrial-scientific-medical (ISM) frequency band in free space is approximately 60 × 60 mm^2^. The antenna size can be reduced to fit into the housing by considering the high dielectric constants of brain tissues (e.g., cortical bone or dura) and by applying antenna miniaturization techniques (e.g., meandering, folding, and shorting). However, most of the reported works suffer from a low antenna gain (<−20 dBi) that is due to the high dielectric loss of brain tissues. The study showed that the antenna gain reduced by 10.7 dB after implanting it in the brain [5].

**Figure 1:**
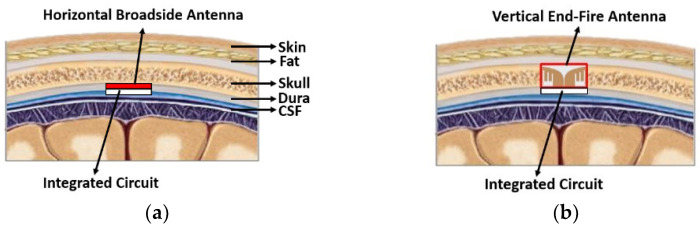
Conceptual illustrations of the brain-implanted antenna placement: (**a**) conventional horizontal placement and (**b**) proposed vertical placement.

Typically, a brain-implanted antenna is located under the skull, immersed horizontally in the dura or cerebrospinal fluid (CSF), as shown in Figure 1a. This circumstance lowers the antenna gain because the skull is thick (~7 mm at the bregma) and lossy (loss tangent, tan*δ*, ~0.3) [14,15]. A full-wave electromagnetic simulation study indicated a reduction of at least 2 dB in antenna gain because of the thick skull. Deformation of the antenna radiation pattern is another issue. A broadside antenna horizontally placed under the skull is expected to radiate toward the zenith. However, unexpected large back lobes and side lobes are often seen after implanting an antenna in such a complex brain environment [16], i.e., the broadside radiation is not guaranteed because of the impact of the thick skull layer, high permittivity brain tissues, and small antenna ground plane.

This paper presents an end-fire radiating antenna implanted vertically in the skull. Figure 1b shows the conceptual illustration of the vertical placement. The antenna was placed along the thickness of the skull and then connected upright to the integrated circuit (IC). Hence, the antenna had more design freedom as long as the height conformed to the thickness of the skull. More importantly, the antenna gain can be significantly improved throughout the bandwidth of interest. The proposed antenna was designed for 3–5 GHz impulse radio ultra-wideband (IR-UWB), which could transfer the data at a high rate with a low power consumption because of its wider operation bandwidth [17,18]. A tapered slot antenna, the so-called Vivaldi antenna, was employed to cater to this wide bandwidth. The Vivaldi antenna is a well-known end-fire radiating antenna that provides broadband impedance matching and radiation performance owing to its gradual tapered structure [19]. However, we found that an abrupt tapering profile was required because the end-fire length was limited to the skull thickness. This abrupt profile limited the end-fire gain and bandwidth. Therefore, a slot array was implemented in the proposed design to resolve the issue. The slot array improved the gain and reduced the antenna resonant frequency, implying the size miniaturization. As a result, the antenna gain is enhanced approximately 2 dB and 3 dB by introducing the new Vivaldi antenna and by implanting the antenna vertically in the skull.

The rest of the paper is organized as follows. Section 2 describes the antenna design. Furthermore, it provides details of the brain environment and slot array structure. Section 3 presents the antenna prototype fabrication process and measurement results. Section 4 discusses the specific absorption rate (SAR) simulation and measurement results along with the link budget analysis to estimate the performance of the communication system equipped with the proposed antenna. Section 5 concludes the paper.

## 2. Antenna Design

**Figure 2:**
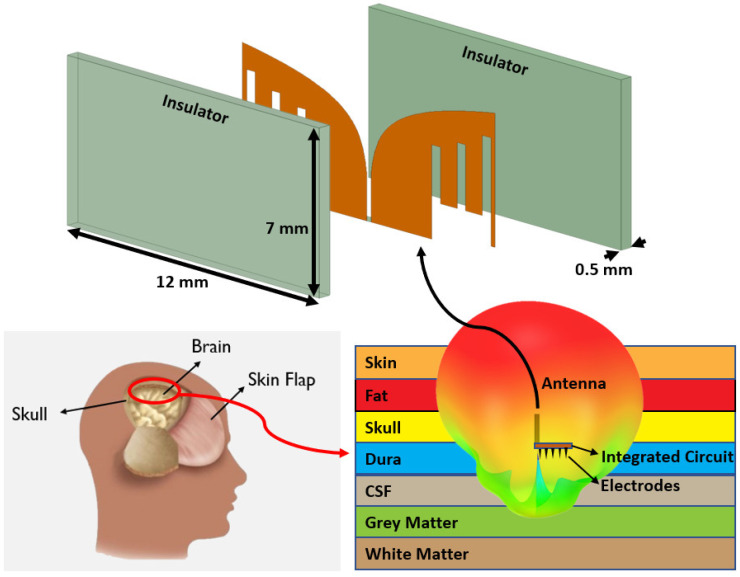
The proposed Vivaldi antenna surrounded by insulators and embedded in the skull.

Figure 2 provides an overview of the proposed skull-embedded Vivaldi antenna. It was vertically installed against the dura matter below the skull. The antenna was sandwiched inside a Taconic RF-35, a biocompatible insulator with relative permittivity (*ε*_r_) and loss tangent (tan*δ*) of 3.5 and 0.002, respectively. Its low tan*δ* value at the antenna design frequency (i.e., 3–5 GHz) is beneficial for improving the antenna gain [11]. In addition, the RF-35 is mechanically durable (tensile strength of 27,000 psi and dimensional stability of 0.00004 mm/mm) and has a low moisture absorption of 0.02%. The height of the insulator was fixed to 7 mm, corresponding to a typical height of an adult human skull. The thickness and width of the insulator were 0.5 mm and 12 mm, respectively (see Figure 2). The width of 12 mm matched half of the guided wavelength (*λ*_g_) at the center frequency (4 GHz). The geometry of the insulated Vivaldi antenna was optimized using full-wave electromagnetic simulation software (Ansys HFSS). The goal was to achieve a good impedance matching condition (reflection coefficient, *S*_11_ < −10 dB) and high end-fire gain (>−15 dB) over a wide bandwidth (3–5 GHz) even in the lossy brain environment. It consisted of seven different layers of brain tissues; their material properties and thicknesses assigned in the simulation model are listed in Table 1 [20]. The material properties of each layer are presented in the center frequency 4 GHz of the target bandwidth (3–5 GHz).

**Figure 3:**
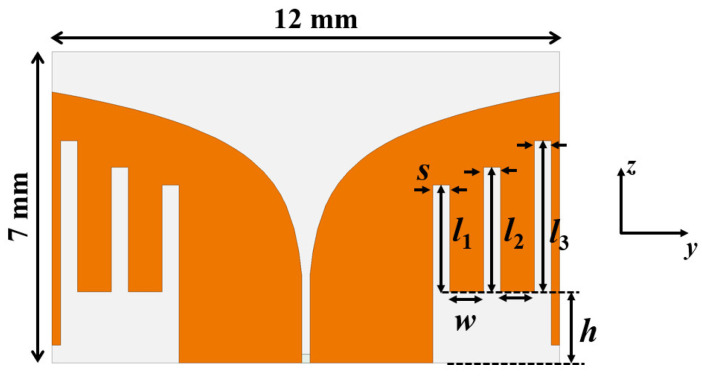
Geometry of the proposed Vivaldi antenna with vertical slots.

The geometry of the Vivaldi antenna is depicted in Figure 3. The tapered slot at the middle gradually opened up to support a smooth impedance transition and to generate end-fire radiations over a broad bandwidth. Simulation results showed that an ordinary Vivaldi antenna modeled in the given area of 7 × 12 mm^2^ resonated around 6 GHz, which is higher than the desirable 4 GHz (i.e., the center frequency of 3–5 GHz). Therefore, an antenna miniaturization technique was required to reduce the antenna footprint. One way to miniaturize a Vivaldi antenna is to add horizontal slots along the side edges [21,22,23]. The horizontal slots act as a choke to lessen undesirable currents flowing at the side edges, which can improve the impedance-matching condition. However, this method accompanies the decrease of end-fire gain. The substrate size along the longitudinal direction was extended [24] and driving elements between the tapered slots were added to recover the end-fire gain [22].

**Figure 4:**
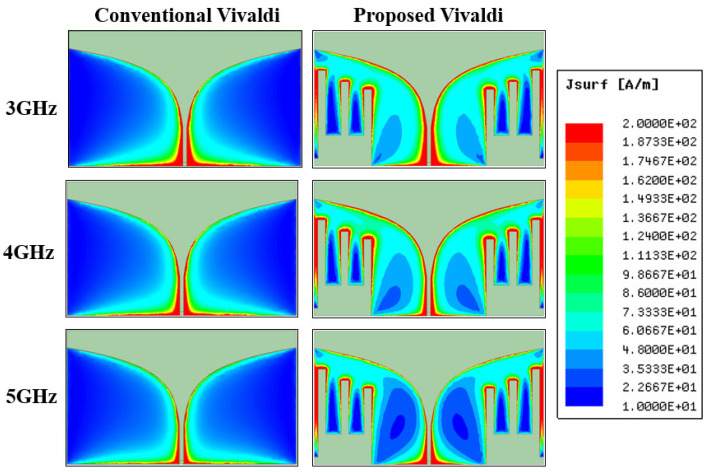
Comparisons of surface current densities of conventional and proposed Vivaldi antennas.

Instead of horizontal slots, the proposed Vivaldi implemented comb-shaped vertical slots to miniaturize the Vivaldi antenna. We found that the method effectively improved the end-fire gain by concentrating more currents along the tapered slot in the middle. Figure 4 shows the surface current densities at 3, 4, and 5 GHz for the conventional Vivaldi without slots and proposed Vivaldi with slots. The conventional design exhibited excessive currents at the bottom edge where the antenna feed was located. Stronger currents were observed at the lower frequency of 3 GHz, which has a longer wavelength. However, the proposed design gradually distributed these currents to the center tapered slot and three vertical slots, making the electrical length longer (miniaturization) and main radiating source stronger (improved gain). The similarity in current distributions at 3, 4, and 5 GHz imply that a stable radiation characteristic can be maintained over a broad bandwidth.

**Figure 5:**
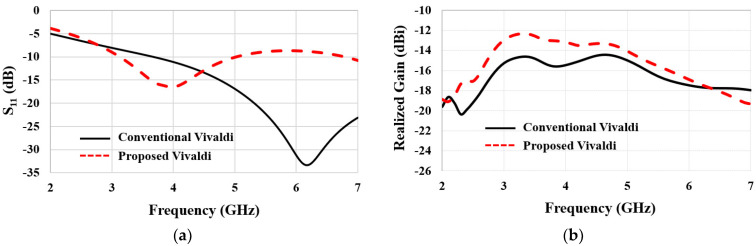
Comparisons of (**a**) *S*_11_ and (**b**) realized gain of conventional and proposed Vivaldi antennas.

Figure 5a,b show the simulation results of *S*_11_ and realized gain while embedding the antenna in the seven-layer brain phantom. We compared *S*_11_ and the realized gain of the proposed Vivaldi antenna to that of the conventional Vivaldi antenna. The resonant frequency was shifted down from 6 GHz to 4 GHz by introducing the vertical slots. Moreover, the realized gain plot showed an improvement of 1–3 dB at the target frequency range, 3–5 GHz; an average of −13 dBi realized gain can be achieved. It is worth noting that the realized gain of a conventional Vivaldi is low, about 6 GHz, despite the *S*_11_ being low, implying its antenna impedance matching and radiation performances have a discrepancy. In contrast, the proposed Vivaldi’s resonant frequency and the frequency exhibiting high realized gain are matched. Altogether, the vertical installation of the end-fire antenna and optimization of its geometry improved the realized gain by approximately 3 dB and 2 dB (total of 5 dB) relative to horizontally installed broadside antennas, respectively.

**Figure 6:**
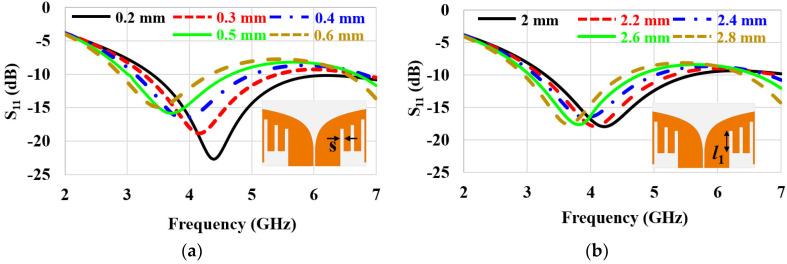
Parametric studies of *S*_11_ by varying (**a**) slot width *s* and (**b**) slot length *l*_1_.

Figure 6 shows parametric studies of *S*_11_ by altering the slot’s geometry. The width of slot (*s*) can be used to tune the antenna’s resonant frequency (see Figure 6a). Here, *s* between each slot is set to be the same to make the optimization process concise. The wider *s* shifted the resonant frequency toward the lower end, resulting in antenna miniaturization by introducing more slot inductance than capacitance. Slot length (*l*_1_) was another parameter for adjusting the resonant frequency (see Figure 6b). The longer *l*_1_ provided more inductance without altering the capacitance. Therefore, the resonant frequency shifted left with increasing *l*_1_. We note that *l*_1_ is the length of the first slot. The second and third slots were longer. Their lengths were determined by the Vivaldi’s exponential curvature profile at the middle. In particular, *l*_1_ = 2.4 mm, *l*_2_ = 2.8 mm, and *l*_3_ = 3.4 mm. Table 2 lists the final antenna’s geometrical parameters. These values were obtained for the target frequency range, 3–5 GHz, with the given antenna space, 12 mm × 7 mm; however, the *s* and *l* parametric optimizations can be applied for any frequency band and antenna size.

## 3. Antenna Prototype Fabrication and Measurement

Having obtained promising broadband and high gain simulation results, an antenna prototype was fabricated and then tested for experimental validation. The measured antenna parameters were *S*_11_, radiation patterns, and realized gain. They were measured by inserting the antenna prototype into an in-house fabricated seven-layer brain phantom.

### 3.1. Fabrication of Brain-Tissue-Emulating Phantom

**Figure 7:**
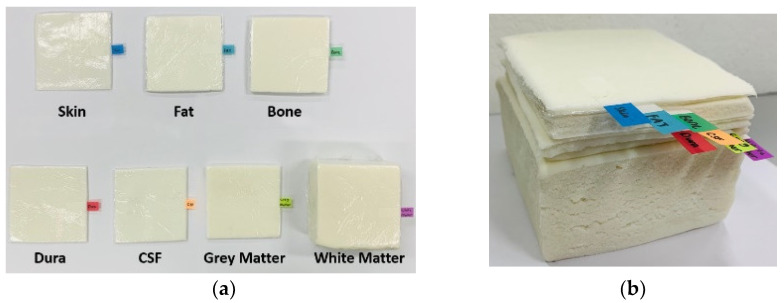
Fabricated brain-tissue-emulating materials: (**a**) pictures of each layer and (**b**) stack-up.

The radiation performance of an implantable antenna is highly affected by its surrounding environment. Hence, it is important to test the antenna inside a human tissue-mimicking phantom exhibiting similar electromagnetic material properties (i.e., *ε*_r_ and tan*δ*) of the actual environment—the brain for this study. A liquid phantom is often used to test implantable antennas [25,26]. However, such a homogeneous phantom is insufficient to represent the complex brain environment consisting of multiple layers with different material properties. We fabricated seven different semi-solid tissue-emulating layers following recipes provided in [12]. Figure 7 shows the fabricated seven layers and their stack-up. The material properties of each layer were measured by an open-ended coaxial probe [27] and compared to the known values listed in Table 1 for validation. The size of the stack-up was 10 cm × 10 cm × 7.2 cm.

### 3.2. Fabrication of Antenna Prototype

**Figure 8:**
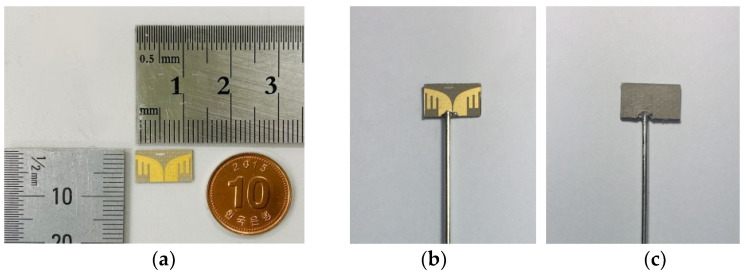
Fabricated antenna prototype: (**a**) antenna printed on a substrate, (**b**) antenna connected to a coaxial cable, and (**c**) antenna covered by a superstrate.

Figure 8 shows the fabricated antenna prototype. The optimized Vivaldi geometry, including the vertical slot array, was implemented on a 0.5 mm thick Taconic RF-35 substrate. A conventional printed circuit board (PCB) fabrication process was used to etch the antenna footprint on the substrate. Figure 8a shows the fabricated antenna itself, while Figure 8b shows a combination of the antenna and coaxial cable. As can be seen, the antenna was directly fed by a coaxial cable instead of a bulky RF connector (e.g., SMA connector). The outer conductor of the cable was directly soldered to one Vivaldi arm to feed the balanced Vivaldi antenna with the unbalanced coaxial cable; the inner conductor was routed through a hole punctured at the substrate and then soldered to the other arm. After that, the antenna was covered by another piece of 0.5 mm Taconic RF-35 as an insulator (i.e., superstrate) as depicted in Figure 8c.

### 3.3. Measurement of S_11_

**Figure 9:**
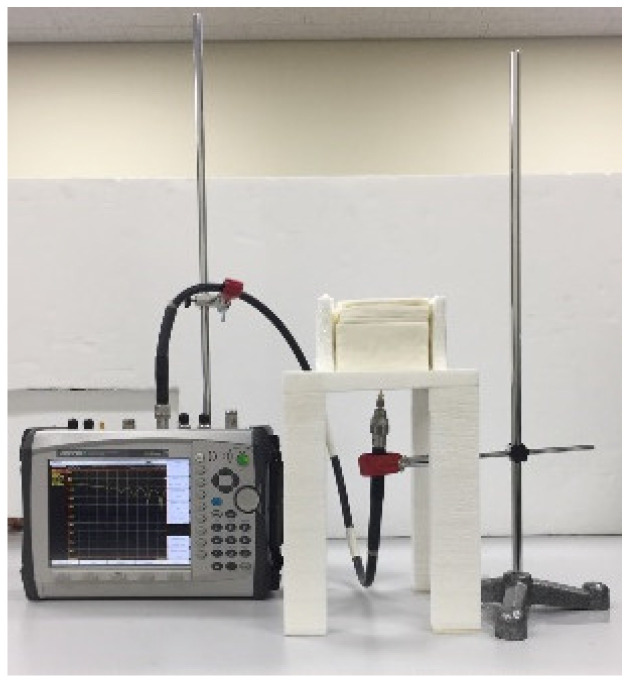
Test setup for *S*_11_ measurement.

**Figure 10:**
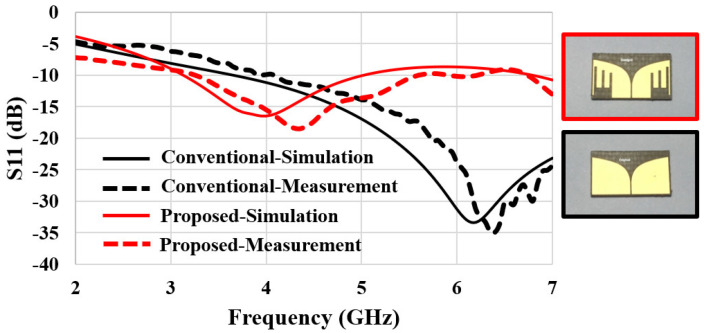
Comparisons of measured and simulated *S*_11_ of conventional and proposed Vivaldi antennas.

The fabricated antenna was placed into the seven-layer phantom for measurements. More specifically, the antenna was vertically inserted in the skull layer; hence, the top and bottom of the antenna were touching the fat and dura layer, respectively (see Figure 2). Figure 9 shows the test setup for *S*_11_ measurement. The phantom (with the antenna inside) was placed on a mount made with low permittivity and low loss Rohacell^®^ foam. The coaxial cable sticking out from the phantom was connected to a vector network analyzer (Anritsu MS2038C). Figure 10 compares the measured and simulated *S*_11_ of the proposed and conventional Vivaldi antennas. The latter is an ordinary Vivaldi that does not have the vertical slot array, as depicted in the inset of Figure 10. The red and black lines correspond to *S*_11_ responses of the proposed and conventional Vivaldi, respectively. The measured *S*_11_ data indicated that the resonant frequency of the proposed Vivaldi was 2 GHz lower than the conventional Vivaldi (4.3 GHz versus 6.3 GHz), which confirms the antenna miniaturization effect caused by the vertical slot array. The simulation results of *S*_11_ are drawn with solid lines. They agree well with the measurements. The measured resonant frequencies were higher than those from simulations by approximately 300 MHz and 150 MHz for the proposed and conventional antennas, respectively, possibly because of slight discrepancies in the phantom’s material properties. We note that the phantom’s *ε*_r_ and tan*δ* are highly affected by the amount of water evaporation with time. It is hard to avoid the water evaporation in spite of precautions when handling the semi-solid phantoms. Solutions to prevent this difficulty can include wrapping each tissue phantom with a thin layer of low permittivity material or using 3D-printed biomaterials with a low moisture content.

### 3.4. Measurement of Radiation Pattern

**Figure 11:**
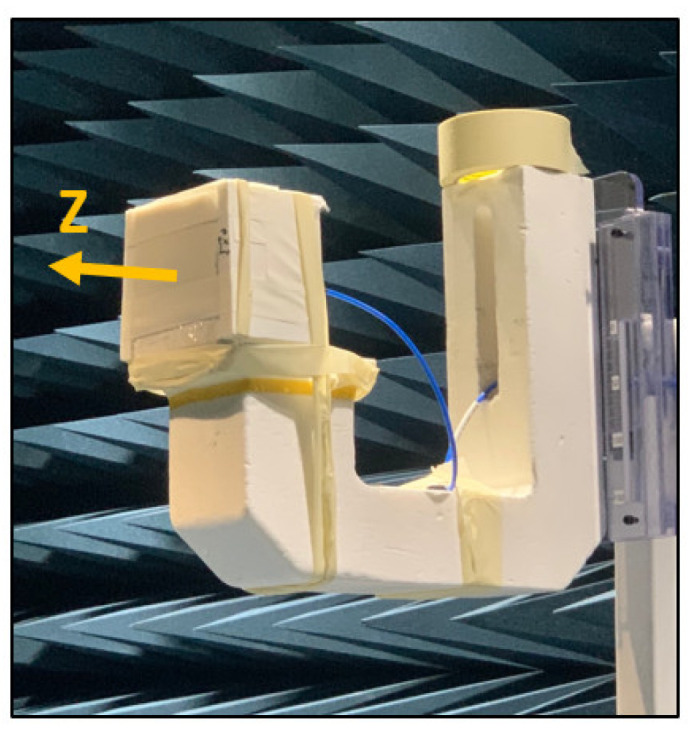
The seven-layer phantom containing the antenna prototype is mounted on the positioner inside the anechoic chamber.

**Figure 12:**
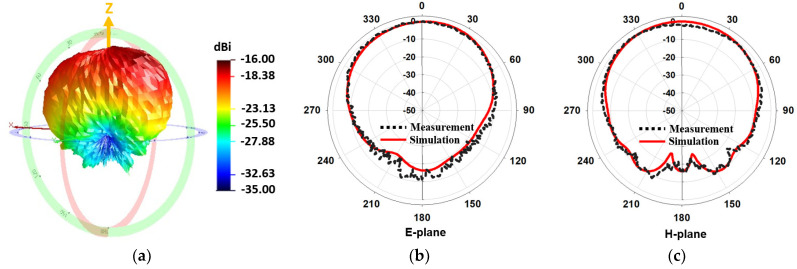
Radiation patterns of the antenna prototype inside the phantom at 4 GHz: (**a**) measured 3D radiation pattern, (**b**) measured and simulated 2D E-plane radiation pattern (normalized by the peak), and (**c**) H-plane radiation pattern (normalized by the peak).

**Figure 13:**
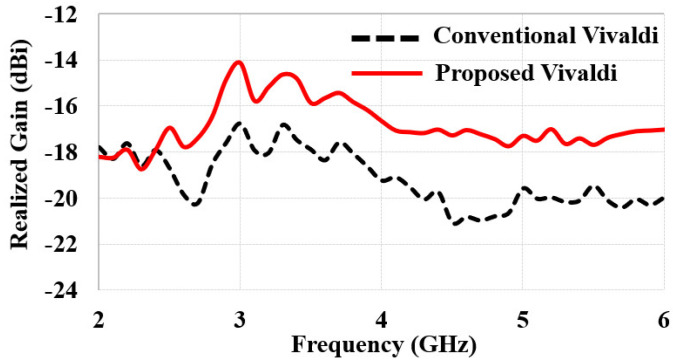
A comparison of measured realized gain of the conventional and proposed Vivaldi antennas.

The far-field radiation patterns of the proposed antenna were measured in an accredited antenna chamber [28]. Figure 11 shows the seven-layer phantom (with the antenna inside) mounted on a positioner. The latter was capable of 3D rotation (180° in elevation and 360° in azimuth). The z-direction shown in the figure corresponds to the end-fire direction where the aperture of the antenna points is. Figure 12a shows the measured 3D radiation pattern at the center frequency, 4 GHz. It shows that most radiation is pointed toward the zenith (z-direction) with a high front-to-back ratio (FBR) of 16 dB. Furthermore, Figure 12b,c present the measured 2D E-plane and H-plane patterns. They were normalized by the peak gain value and then compared with the simulated radiation patterns. Good agreements can be observed, implying the prototype fabrication and measurement procedures were valid. Figure 13 shows the measured realized gain in the z-direction at the frequency range of 2 to 6 GHz. We compared the measured realized gain of the conventional Vivaldi (see Figure 10 b) with that of the proposed Vivaldi (see Figure 10). The measured realized gain data was not steady in the bandwidth of interest, and it was approximately 2 dB lower than the simulation data (see Figure 5b); however, the gain improvement of the proposed design compared to the conventional Vivaldi was clearly observed. A gain improvement is observed because of the added vertical slots, which redirect more currents along the tapered slot as described in the simulation study, Figure 4. The average improvement was 2.68 dB throughout 3–5 GHz, which is similar to the improvement demonstrated in Figure 5b. Table 3 compares previously reported brain-implanted antennas and the proposed Vivaldi in terms of their operation frequency, size, and gain. It also includes the conventional Vivaldi’s gain to highlight the gain improvement of the proposed Vivaldi.

## 4. Specific Absorption Rate and Link Budget Analysis

### 4.1. Specific Absorption Rate

It is required to examine the specific absorption rate (SAR)—the amount of non-ionizing radiated power absorbed by the surrounding biological tissues—for an implantable wireless device. SAR standards differ by countries or regulatory agencies; however, two SAR standards are mainly considered: IEEE C95.1-1999 [29] and IEEE C95.1-2005 [30]. The maximum allowable SAR values are 1.6 W/kg averaged over 1 g of tissue and 2 W/kg over 10 g of tissue for IEEE C95.1-1999 and IEEE C95.1-2005, respectively.

**Figure 14:**
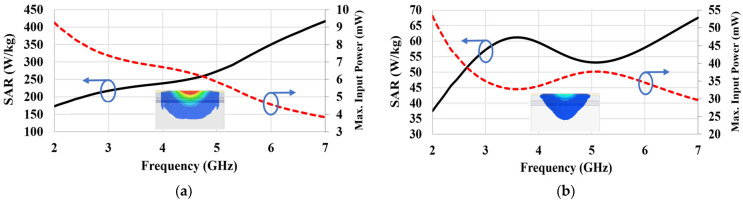
The simulated SAR and maximum allowable input power of the proposed antenna inside the seven-layer phantom: (**a**) SAR-1g and (**b**) SAR-10g. The insets show simulated SAR value distributions around the antenna.

SAR values were computed and analyzed using the same simulation setup as the antenna performance analysis (see Section 2). Figure 14a,b show the simulated SAR-1g and SAR-10g for various frequencies after supplying an input power of 1 W to the antenna embedded in the bone layer. The SAR-1g in Figure 14a continuously increased with increasing frequency, while the SAR-10g in Figure 14b shows a peak and null at 3.6 GHz and 5.1 GHz, respectively. The SAR values were very high because of the high input power of 1 W, e.g., 240 W/kg and 59.4 W/kg at 4 GHz for SAR-1g and SAR-10g, respectively. However, the actual SAR is expected to have a much lower value because the power consumed by the RF-front-end of implantable devices generally lies within 100 μW to a few mW [31]. Hence, we calculated the maximum allowable input power to the antenna that satisfied the SAR-1g (1.6 W/kg) and SAR-10g (2 W/kg) criteria. They are marked by the red dashed lines. The SAR-1g criterion can be fulfilled as long as the input power is less than 5.9 mW for the frequency range of 3–5 GHz as in Figure 14a. The maximum allowable input power for the SAR-10g was 33.6 mW as in Figure 14b, which is less stringent than SAR-1g.

**Figure 15:**
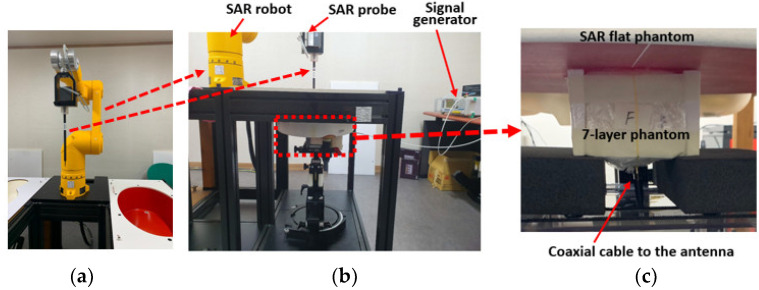
SAR measurement setup with DASY 5 SAR robot: (**a**) SAR robot with the probe, (**b**) whole measurement setup, and (**c**) antenna placement.

**Figure 16:**
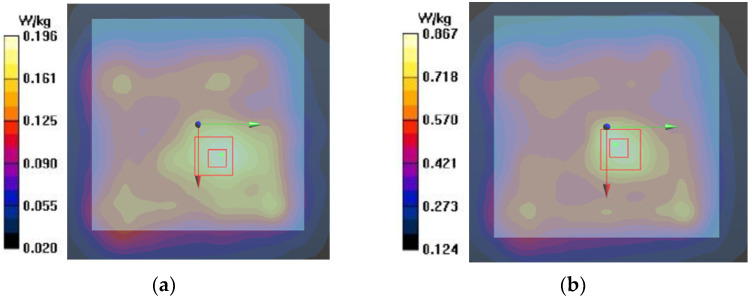
Measured SAR distribution: (**a**) 2.45 GHz and (**b**) 5.8 GHz. Red boxes indicate the hot spot.

We measured SAR using a SAR robot in an accredited test facility [32]. Figure 15a shows the SAR robot and probe and Figure 15b shows the actual test set-up with the phantom and signal generator as the source. Figure 15c provides a zoomed-in view of the phantom placements. The seven-layer phantom with the antenna inside was attached at the bottom of a SAR flat phantom. A SAR probe was scanned by the robot arm at the opposite side of the empty flat phantom, and the E-field magnitudes radiated from the antenna were collected. The antenna was fed by a coaxial cable connected to a signal generator. The SAR values at a wireless local area network (WLAN) of 2.45 GHz and 5.8 GHz were measured because of the lack of SAR measurement procedure for 3–5 GHz IR-UWB. The output power from the signal generator was set to 1 mW. Figure 16 shows the measured SAR distribution. The total scan area was 150 × 150 mm^2^. The zoomed scan volume after identifying the hot spot was 40 × 40 × 35 mm^3^. The hot spot locations for 2.45 GHz and 5.8 GHz were comparable. The higher frequency (5.8 GHz) showed a higher averaged SAR-1g value of 0.42 W/kg than that of the lower frequency (2.45 GHz) (i.e., 0.11 W/kg). Both of them were much lower than the 1.6 W/kg limit. These measurement trials provide a reasonable postulation that SAR is not a problematic issue despite that the measured frequencies were not exactly matched to the target frequencies.

### 4.2. Link Budget Analysis

A link budget analysis [33,34] was conducted to estimate the approximate performance of an implantable wireless communication system equipped with the proposed Vivaldi antenna. We assumed a point-to-point wireless communication system whose transmitting antenna was the proposed Vivaldi implanted in the brain and the receiving antenna was a broadband testing antenna [35] situated outside of the head. Table 4 summarizes the parameters for the link budget analysis. The link margin (LM) is a power margin at the receiver, allowing a satisfying wireless communication quality. More specifically:

(1) $$LM\left(dB\right)=P_{r}-P_{r\left(min\right)}$$

where *P*_r_ corresponds to the received power and *P*_r(*min*)_ denotes the minimum required power for the receiver. *LM* typically spans from 3 to 20 dB. We set it to 20 dB, the most demanding requirement, to reflect the harsh wireless communication environment. The assigned transmit power (*P*_t_) was −25 dBm, which is a typical output power of a transmitter for implantable devices [31]. The realized gain of the transmitting (Tx) antenna (i.e., implanted antenna) was given by −16.67 dB based on the measurement in the data presented in Section 3. Furthermore, the receiving (Rx) antenna’s realized gain was set to 6.65 dB, which corresponds to the antenna gain at 4 GHz of a broadband tapered slot antenna [35]. The required signal-to-noise ratio (SNR) per bit, or the energy per bit to noise power spectral density ratio (*E*_b_/*N*_0_), was set to 9.6 dB by assuming an ideal phase-shift keying (PSK) performance. The bit rate was set to 256 Mbps, which is reasonably high for brain-signal monitoring [34]. Finally, the path loss (*L*_0_) was calculated for the free-space attenuation. In the equation, *λ*_4GHz_ denotes the free-space wavelength of 4 GHz (i.e., 75 mm) and *D* denotes the distance between the Tx and Rx. In fact, *D* is the parameter of interest for this link budget analysis. According to the Friis transmission formula [33]:

(2) $$P_{r}\left(dB\right)=P_{t}+RG_{t}+RG_{r}-L_{0}$$

(3) $$P_{r\left(min\right)}\left(dB\right)=\frac{E_{b}}{N_{0}}+KT_{0}+B$$

Plugging (2) and (3) into (1) and then applying the parameters from Table 4 provided *D* = 108.1 mm, i.e., the transmitted brain signal can be reliably received with *LM* of 20 dB at the receiver located at 108.1 mm above the head. We note that this distance was reduced to 80.4 mm if the conventional Vivaldi with a realized gain of −19.24 dB was used instead of using the proposed Vivaldi.

## 5. Conclusions

The full-wave electromagnetic simulations showed that the end-fire antenna vertically embedded in the skull exhibited two times (3 dB) higher antenna gain than an ordinary case—a broadside antenna horizontally embedded below the skull. The proposed end-fire Vivaldi antenna was measured to have a small footprint of 12 × 7 mm^2^ because of the novel comb-shaped slot arrays behind the main aperture. These slot arrays not only promote the antenna miniaturization but also enhance the gain and bandwidth. The geometry of the antenna was carefully optimized by full-wave simulations to operate in the 3–5 GHz IR-UWB frequency range. An antenna prototype was fabricated and a series of measurements were performed by embedding the antenna in an in-house-made seven-layer brain-tissue-emulating phantom to verify the antenna performance. The measurement results of the proposed Vivaldi showed that the resonant frequency was 2 GHz lower, and the gain was 2.6 dB higher than the conventional Vivaldi without slot arrays. Furthermore, the proposed Vivaldi can be a promising candidate for brain-to-outside wireless communication based on the link budget and SAR analyses. Based on our findings, it could be worthwhile for surgeons and medical professionals to identify pathways for implanting antennas in the skull during a brain surgery (e.g., craniotomy).

## Figures and Tables

**Table 1:** Material properties of brain tissues at 4 GHz.

Layer	Relative Permittivity (*ε*_r_)	Loss Tangent (tan*δ*)	Thickness (mm)
Skin	40.84	0.297	1
Fat	5.12	0.160	2
Skull	10.53	0.310	7
Dura	40.10	0.308	1.5
CSF	63.73	0.366	2

**Table 2:** Optimized antenna parameters.

Parameter	Length (mm)
*s*	0.4
*w*	0.8
*h*	1.6
*l*_1_	2.4
*l*_2_	2.8
*l*_3_	3.4

**Table 3:** Comparison of brain-implanted antennas.

Antenna	Frequency (GHz)	Size (mm^3^)	Gain (dBi)
[11]	2.40–2.48	39.9	−20.75
[12]	2.42–2.50	50	−25
[14]	2.40–2.48	101.6	−17.3
Conventional Vivaldi	3–5	42	−18.3
Proposed Vivaldi	3–5	42	−15.7

**Table 4:** Parameters for link budget analysis.

Parameters	Values at 4 GHz
Link margin (*LM*)	20 dB
Transmit power (*P*_t_)	−25 dBm
Tx Ant. realized gain (*RG*_t_)	−16.67 dB
Rx Ant. realized gain (*RG*_r_)	6.65 dB
SNR per bit (*E*_b_/*N*_0_)	9.6 dB
Boltzmann’s constant (*K*)	1.38 × 10^−23^
Temperature (*T*_0_)	298 K
Bit rate (*B*)	256 Mbps
Path Loss (*L*_0_)	$20\log\left(\frac{4\pi D}{\lambda_{4\mathrm{GHz}}}\right)\mathrm{dB}$

## Data Availability

The data presented in this study are available on request from the corresponding authors.
